# A Young Female With Takayasu Arteritis Involving the Coronary Arteries

**DOI:** 10.7759/cureus.53927

**Published:** 2024-02-09

**Authors:** Jasneet Singh, Suman Jatain, Mahendra Chouhan, Gurpreet Singh, Devika Gupta

**Affiliations:** 1 Cardiology, Janakpuri Super Speciality Hospital, Delhi, IND; 2 Critical Care Medicine, Janakpuri Super Speciality Hospital, Delhi, IND; 3 Neurology, Janakpuri Super Speciality Hospital, Delhi, IND

**Keywords:** cad in young female, aortitis, cardiology, radiology, angiography, large vessel vasculitis, cad: coronary artery disease, takayasu arteritits

## Abstract

A rare form of large vessel vasculitis, Takayasu arteritis (TA) typically affects the aorta and its primary branches and rarely involves the coronary arteries. We present a case study of a female patient who had refractory hypertension and coronary artery disease for which she underwent percutaneous transluminal angioplasty. Subsequently, she was diagnosed with Takayasu arteritis. We wish to underscore the significance of timely identification and intervention as pivotal factors in improving patient outcomes and optimizing the effectiveness of therapeutic strategies in managing TA.

## Introduction

Takayasu Arteritis (TA) is a rare type of inflammation of blood vessels and was first documented in 1908 in a Japanese individual who exhibited abnormalities in their retinas [1]. This is a complex, long-lasting condition characterized by inflammation, primarily impacting young female individuals [2-4]. The diagnosis of TA can be difficult as specific biomarkers are not present. However, detailed medical history and clinical examination, as well as the assessment of inflammatory markers such as erythrocyte sedimentation rate(ESR) and C-reactive protein(CRP), can help in making a diagnosis. Additionally, vessel inflammation can be identified using computed tomography(CT) and magnetic resonance imaging (MRI) [5].

The revised 2022 American College of Rheumatology criteria for TA requires the patient to score a minimum of five points to be classified as TA. The patient’s age should be below 60 years and must show evidence of vasculitis on imaging. Additionally, points are awarded for meeting either clinical or imaging criteria. In the clinical field, one point is assigned for being female and a difference of more than 20 mmHg in systolic blood pressure in arms. Two points are given if the patient has angina, claudication, bruit, reduced or absent pulse, and abnormalities in the carotid arteries. The imaging criteria gives points according to the number of affected arterial sites [6].

The best way to assess mural inflammation or vessel lumen changes is through MRI; the lack of radiation risks and reliable detection of active wall inflammation make it preferable. Young patients under 40 with TA are mostly females and show involvement of the thoracic aorta, while males show involvement of the abdominal aorta and branches. Although the exact pathogenesis is uncertain, some studies indicate that mast cells and cytotoxic lymphocytes form large amounts of a granulomatous inflammatory infiltrate, thus damaging the vessel by producing fibrotic tissue that leads to the lesions causing TA [7].

The inflammatory process in TA causes the vessel walls to become hyperplastic and thicken, leading to ischemia. Inflammation is mostly segmental and can cause stenosis, occlusion, or dilation. It can affect a wide variety of vessels including the subclavian, brachiocephalic trunk, carotid, vertebral, and abdominal aorta [8]. Only 9-11% of TA patients show coronary artery involvement, mostly in the coronary ostia. One-fifth of TA patients have critical vascular lesions and are refractory [9].

We describe a case of a female patient diagnosed with Coronary Artery Disease(CAD) with TA. Our goal is to provide information on the significance of coronary involvement in large vessel vasculitis, which may alert medical professionals to the need for prompt assessment to initiate appropriate therapy as soon as possible.

## Case presentation

A 37-year-old Indian female patient was referred to the cardiology outpatient with uncontrolled hypertension. A detailed history indicated that the patient had angina on exertion class 2 with malaise and fatigue. Upon evaluation, she was afebrile and had stable vitals, had no bruit, and her cardiac examination was normal. The left arm's blood pressure was measured at 150/77 mm Hg, but the right arm's was higher at 179/95 mm Hg. Additionally, the left radial artery palpation revealed a weak and thready pulse as compared to a full and bounding pulse on the right radial artery palpation.

Electrocardiogram (ECG) showed a left ventricular hypertrophy(LVH) strain pattern with down-sloping asymmetrical ST-segment depression with an inverted asymmetric T wave. Her white blood cells were within the normal range, whereas CRP and ESR were elevated. Echocardiography revealed moderate concentric left ventricular hypertrophy without any wall motion abnormalities, and a normal aortic valve with an estimated ejection fraction of 55%. The patient was taken up for a planned coronary and peripheral angiography.

Coronary angiography revealed a porcelain aorta (heavily calcified aorta), left anterior descending artery (LAD) showing 70% stenosis proximally with ectasis, left circumflex artery (LCX) showing about 90% stenosis distally, right coronary artery(RCA) showing 60% stenosis in middle segment, and a dilated and large left main coronary artery. Peripheral angiography revealed multiple stenosis in the right subclavian artery and approximately 80% stenosis in the bilateral renal arteries; the left subclavian artery was not visualized. Subsequently, MR angiography revealed complete thrombosis of the left subclavian and left common carotid arteries. There was also about 80-90% luminal narrowing of the long segmental lower thoracic and abdominal aorta. These findings suggested the diagnosis of Takayasu Vasculitis as the 2022 ACR score was 12. The patient subsequently underwent percutaneous angioplasty with drug-eluting stents in the LAD and LCX coronaries and was put on dual antiplatelet therapy.

**Figure 1:**
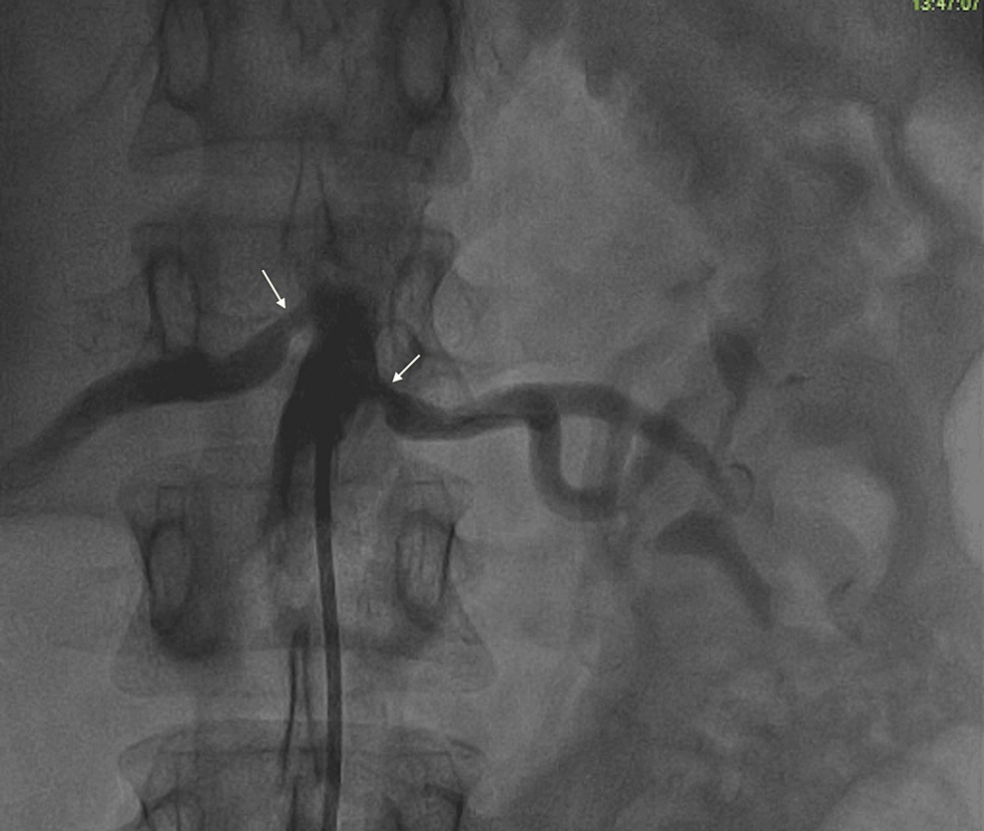
Peripheral angiography showing bilateral renal artery stenosis

**Figure 2:**
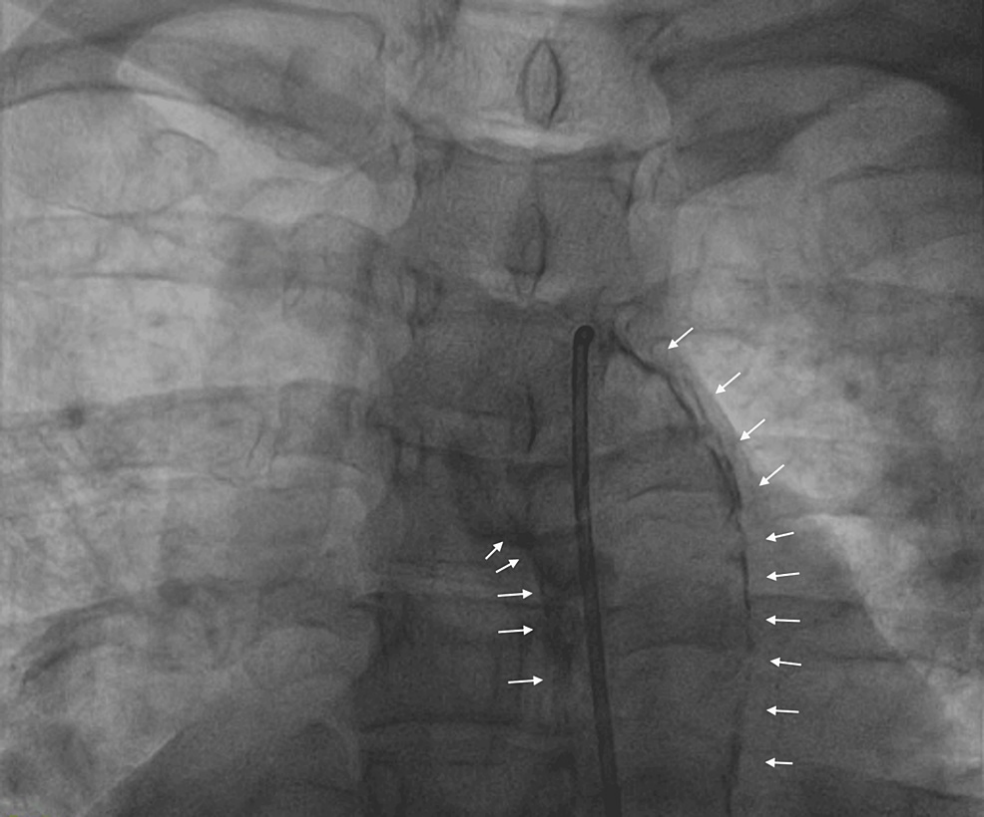
Heavy calcification seen in the arch of the aorta

**Figure 3:**
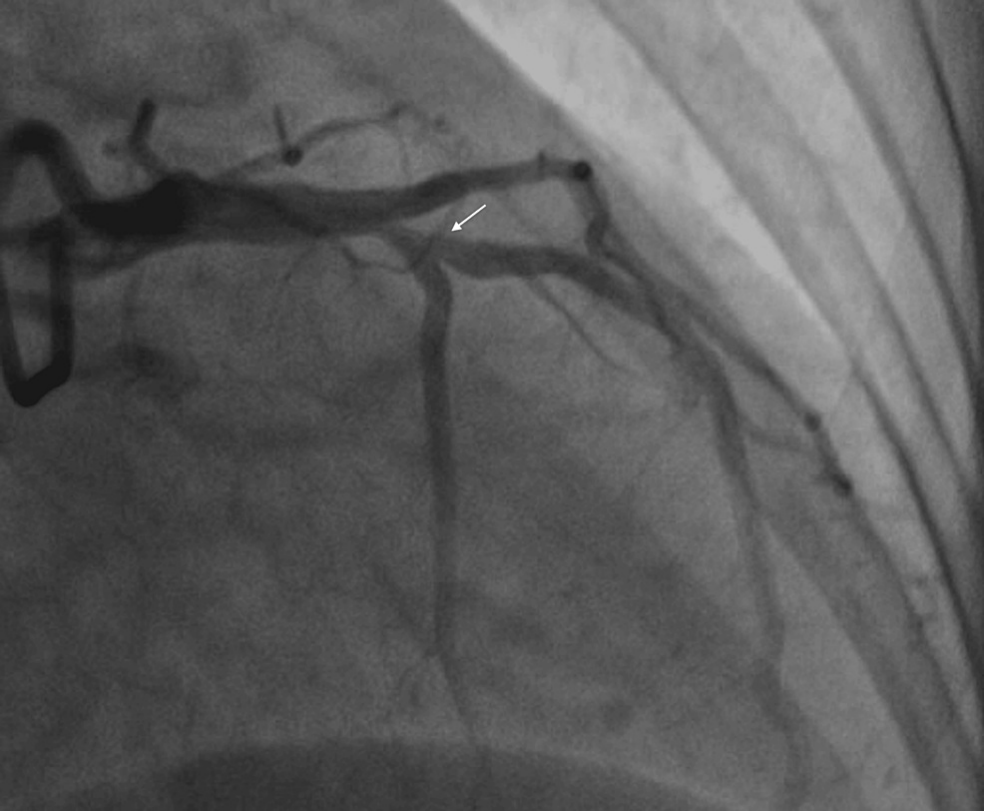
Coronary angiography showing proximal stenosis in the LAD LAD: Left Anterior Descending Artery

## Discussion

CAD is rarely observed in young women, but it poses an important challenge for both patients and physicians due to its severe impact on the lives of young patients [10]. Our patient visited the outpatient department multiple times complaining of chest pain. However, because her predictive risk scores were low, she was discharged without any additional investigation. Finally, she was referred to cardiology outpatient services for uncontrolled hypertension. Misdiagnosis frequently occurs in patients who do not have CAD related to atherosclerosis, such as those with autoimmune diseases.

TA is characterized as pan-arteritis causing ischemic symptoms related to stenotic lesions [11]. The patient had problems with both the aorta and the coronary arteries, but they only had symptoms related to CAD and not any other symptoms, which made the diagnosis harder. TA usually gets worse over time and returns. It can affect the coronary arteries, the aorta, and the subclavian arteries, and have terrible long-term consequences.

Takayasu arteritis should be diagnosed early during the prestenotic phase. Clinical features and imaging are key to diagnosis. Acute phase reactants, such as ESR and CRP, may show elevated levels, but they may not accurately indicate disease severity and may appear normal even when active [12]. Several imaging modalities, including high-resolution ultrasound, magnetic resonance imaging (MRI), magnetic resonance angiography (MRA), CT angiography (CTA), and positron emission tomography (PET), have been studied in patients with TA. The currently available limited and usually retrospective studies indicate that while no single method can provide all the necessary information, they may have distinct and complementary functions in patient care [13,14]. During the stable phases of the disease, the prognosis of percutaneous coronary intervention (PCI) is comparable to that of coronary artery bypass graft (CABG) surgery. Recent data indicate that both types of interventions have a high rate of failure [15], with reported mortality ranging from 3% to 21.0% [16].

This report demonstrates the intricacy involved in diagnosing and treating TA with CAD. Young women without classical risk factors should be evaluated for the presence of TA. The most effective treatment should be determined based on coronary anatomy and age. To prevent early unfavorable outcomes, it is crucial to maintain appropriate follow-up and immunosuppressive therapy.

## Conclusions

The nonspecific symptoms of TA can conceal significant vascular damage, making diagnosis difficult. The case presentation vividly illustrates the clinical manifestations, diagnostic workup, and subsequent interventions in a 37-year-old female patient with CAD associated with TA. The patient's journey, from initial complaints of chest pain which were ignored, to the discovery of a heavily calcified aorta and multiple arterial stenosis, serves as a valuable clinical narrative.

The case reinforces the need for vigilant evaluation of young individuals with atypical symptoms, as misdiagnosis may lead to delayed intervention and adverse outcomes. In female patients with early diffuse vascular disease, clinicians should consider TA. It can be practically screened by measuring arterial blood pressure in both arms. CT and MR angiography are suitable assessment methods. As immunosuppression may slow disease progression, early diagnosis is crucial. Early diagnosis in the presented case could have significantly benefited the patient by enabling timely initiation of immunosuppressive therapy, which could have potentially impeded the disease progression, preventing further vascular damage.
